# When Walking Outlasts Cognition: Rethinking the Late-Stage of Lewy Body Dementia

**DOI:** 10.1159/ndd/aduag001

**Published:** 2026-08-13

**Authors:** Patrick Stancu, Anna E. Goodheart, Lisa Hentsch, Caroline Hertler, Gilles Allali, Frédéric Assal

**Affiliations:** a Division of Neurology, Department of Clinical Neurosciences, Geneva University Hospitals, Geneva, Switzerland; b Department of Neurology, Massachusetts General Hospital, Boston, MA, USA; c Harvard Medical School, Boston, MA, USA; d Division of Palliative Medicine, Department of Rehabilitation, and Geriatrics, Geneva University Hospitals, Geneva, Switzerland; e Competence Center for Palliative Care, Department of Radiation Oncology, University Hospital and University of Zurich, Zurich, Switzerland; f Leenaards Memory Center, Department of Clinical Neurosciences, Lausanne University Hospital and University of Lausanne (CHUV-UNIL), Lausanne, Switzerland

**Keywords:** Gait, Lewy body, Parkinson’s disease, Alzheimer’s disease, Supportive care

## Abstract

**Background:**

Lewy body dementia (LBD) is characterized by pervasive cognitive, motor, and neuropsychiatric symptoms. Although cognitive decline has traditionally shaped staging and prognosis, accumulating evidence indicates that gait and postural disturbances follow partially independent trajectories and may remain preserved even as cognition deteriorates. These observations challenge conventional assumptions about late-stage decline and suggest that locomotor function may complement cognitive measures when assessing late-stage transition in LBD.

**Summary:**

This review synthesizes current evidence on gait disturbances across the LBD spectrum and their neural substrates, in comparison with Alzheimer’s disease (AD) and Parkinson’s disease (PD). Gait impairment in LBD arises from distributed network dysfunction involving dopaminergic and cholinergic pathways, fronto-striatal, parietal, and cerebellar contributions, resulting in greater gait variability, reduced automaticity, and heightened dual-task costs relative to AD and PD. Despite this vulnerability, preserved procedural memory, brainstem locomotor circuits, and cerebellar-cortical compensation may allow walking to persist longer than cognition in some individuals. In advanced disease, rapid gait deterioration has been described near the end of life, suggesting that gait trajectories may capture aspects of late-stage decline that cognitive measures, affected by fluctuations and attentional instability, may not reliably reflect.

**Key Messages:**

Gait and cognition do not decline in parallel in LBD; in some individuals, walking may outlast cognitive abilities. Gait disturbances reflect distributed network dysfunction rather than isolated motor system degeneration. In late stages, gait performance may offer a clinically useful indicator of disease transition when cognitive testing becomes less reliable. Integrating gait assessment into clinical evaluation may enhance prognostication and support palliative care planning in LBD.

## Introduction

Gait and postural deficits are common in Lewy body dementia (LBD), but their underlying mechanisms are less well characterized than the cognitive and neuropsychiatric manifestations [1]. These motor disturbances arise within a complex neurodegenerative landscape, as LBD, the second most common neurodegenerative dementia after Alzheimer’s disease (AD), encompasses both dementia with Lewy bodies (DLB) and Parkinson’s disease dementia (PDD) [2, 3]. Both are closely related clinical syndromes sharing a common neuropathology, characterized by widespread alpha-synuclein accumulation in Lewy bodies and neurites, with degeneration of dopaminergic midbrain and cholinergic basal forebrain neurons [4, 5]. AD co-pathology is frequently observed [6]. Clinically, these disorders include progressive cognitive impairment associated with parkinsonism, sleep disturbances, visual hallucinations, and fluctuations of attention and wakefulness [7]. Ultimately, the distinction between DLB and PDD relies on the timing of cognitive and motor symptom onset: DLB is diagnosed when dementia occurs before or within 1 year of the onset of parkinsonism, whereas PDD requires motor symptoms to precede cognitive decline by more than a year [8].

Historically, gait disturbances in LBD have been primarily attributed to the dopaminergic neurodegeneration underlying parkinsonism, which leads to bradykinesia, reduced stride length, and slower walking [9]. Yet, dopaminergic loss alone does not fully account for the complexity or variability of gait impairments observed in LBD [10]. Converging neuroimaging and neuropathological findings indicate that gait regulation depends on a wider network of systems [9]. Non-dopaminergic pathways, particularly cholinergic circuits [11], along with cortical and subcortical network alterations [12] and cerebellar involvement [13] contribute to gait control beyond the basal ganglia and mesencephalic locomotor region [14]. As cholinergic deficits emerge as contributors to gait impairment, clinical studies are evaluating whether enhancing this system is beneficial [15]. Pilot and randomized trials in PD and AD indicate that cholinesterase inhibitors can improve gait stability and reduce falls [16, 17]. In addition, white matter disconnection [18] and impaired visuospatial integration have been linked to deficits in gait and balance [9]. Together, these mechanisms highlight the distributed neural control of gait, which may help explain the heterogeneity of gait disturbances in LBD and their distinction from those observed in AD and cognitively normal Parkinson’s disease (PD) [19]. Beyond its neurobiology, gait has emerged as a sensitive indicator of disease progression: quantitative gait analysis can predict cognitive decline and conversion to dementia and may also help differentiate between dementia subtypes, underscoring its potential as a marker for diagnosis and prognosis [20]. Because gait is a complex cognitive-motor function relying on multiple brain regions, its impairment may reflect early neurodegenerative changes in mild dementia and progressive deterioration in later phases within the LBD spectrum [6, 21].

The aims of this review are to synthesize current evidence on gait disorders and their neural correlates across LBD, PD, and AD and to examine the evolution and clinical significance of gait changes in late-stage disease. We further consider whether, in some cases of LBD, gait measurements could serve as clinical markers of late-stage dementia, complementing cognitive measures in assessing disease prognosis and signaling end-stage transition. This narrative review was informed by a targeted PubMed search published from 1990 through November 2025, using combinations of the terms “Lewy body dementia,” “dementia with Lewy bodies,” “Parkinson disease dementia,” “Parkinson disease,” “Alzheimer disease,” “gait,” “dual-task walking,” “supportive care,” and “palliative care.” We prioritized original studies, longitudinal cohorts, neuroimaging and neuropathologic studies, and clinically focused reviews addressing gait phenotypes, cognitive-motor interactions, and disease progression across the LBD spectrum and related disorders.

## Gait and Cognition in LBD and Related Neurodegenerative Disorders

### Comparative Gait Phenotypes across LBD, PD, and AD

Gait, balance, and motor impairments are common across dementias and contribute to falls, functional decline, and avoidable hospitalizations [22]. Diagnostic accuracy is often challenged by overlapping clinical and neuropathological features along the PD-PDD-DLB-AD spectrum, which can blur conventional disease boundaries. Nonetheless, gait and motor performance show distinct patterns across these disorders, with characteristic profiles in LBD, PD, and AD (shown in Table 1) [23, 24]. Nevertheless, the motor phenotype of cognitively normal PD differs from that observed in other parkinsonian disorders such as DLB and PDD, reflecting distinct underlying pathophysiological mechanisms [24]. In a clinically matched study, individuals with LBD showed more pronounced axial motor, gait, and balance impairments than those with PD despite similar total motor scores and performed worse than AD subjects on all motor measures [25]. Moreover, comparative gait analyses in individuals with mild-to-moderate disease severity show that gait impairments are least severe in AD, more pronounced in PD, and most severe in LBD [26]. Fritz et al. [26] found that individuals with LBD walk more slowly than those with PD, with shorter stride lengths, longer stance times, and increased spatial and temporal gait variability during comfortable and fast walking. Compared with AD, patients with LBD display marked slowing, reduced stride length, and abnormal swing-stance ratios during fast forward and backward walking, as well as significantly higher gait variability across all walking conditions [26, 27]. Balance deficits are also more pronounced in LBD than in AD or PD [25]. Consistently, axial postural instability and gait abnormalities correlate most strongly with executive and visuospatial deficits, whereas non-axial motor features such as tremor and limb rigidity do not differentiate LBD from PD [25, 28].

**Table 1.** Comparative clinical and gait characteristics across DLB, PDD, PD, and AD at disease diagnosis

​	DLB	PDD	PD	AD
Onset	Cognitive decline occurs before or within 1 year of motor onset	Motor impairment precedes cognitive decline by more than a year (“1-year rule”)		Cognitive decline precedes subtle gait changes
Course of disease	Fluctuating; gradual decline	Gradual decline In PDD, dementia usually occurs more than 2 years after motor symptoms (40–78%)		Gradual decline
Motor profile	Less frequent rest tremor; more prominent postural instability	Phenotype-related – Tremor-dominant: ↓ progression rate; mild postural instability – PIGD: ↑ progression rate; early gait disturbance and postural instability; ↑ risk of dementia (PDD)		Gait disturbances and rigidity in later stages only
Gait domains				
Pace	↓↓ Velocity and stride length	↓↓ Velocity; ↓↓↓ stride length	↓ Velocity and stride length	Slight ↓ velocity; stride length relatively preserved
Rhythm	↓ Regularity of step timing	↓↓ Step regularity; festination, and irregular turning frequent	Slight ↓ regularity; irregular turning and festination in more advanced stages	Minimal changes of rhythm
Variability	↑↑ Stride-to-stride variability; ↑ under dual-task interference	↑↑↑ Stride-to-stride variability; ↑↑ under dual-task interference	↑ Stride-to-stride variability; ↑ under dual-task interference	↑ Stride-to-stride variability, increasing with cognitive decline; ↑ under dual-task interference
Asymmetry	Mild	Marked asymmetry	Marked asymmetry	Symmetric gait
Postural control/balance	↓↓ Stability; ↑↑ falls	↓↓↓ Stability; ↑↑ falls	↓ Stability; ↑ falls	Mild ↓ stability (cognitive-related)
Dual-task interference	↓↓ gait speed and stability, freezing occasionally	↓↓ gait speed and rhythm; freezing frequent	↓ gait speed & ↑ variability	↓ Gait speed and ↑ variability
Neuropathology	Widespread alpha-synuclein pathology and AD co-pathology of variable severity and extent	Advanced alpha-synuclein pathology. AD co-pathology present but less severe and extended in PDD.	Midbrain alpha-synuclein pathology	Beta-amyloid plaques, neurofibrillary tangles

Arrows indicate the direction and relative magnitude of change compared with healthy controls, with ↑ indicating increase and ↓ indicating decrease. Classification based on evidence synthesized from previously published studies [25, 26, 29–31].

DLB, dementia with Lewy bodies; PDD, Parkinson’s disease dementia; PD, Parkinson’s disease; AD, Alzheimer’s disease; PIGD, postural instability and gait difficulty.

### Gait Phenotypes within the LBD Spectrum

Comparing gait characteristics between cognitively normal PD, PDD, and DLB remains challenging, owing to differences in age at onset and disease duration, variable dopaminergic treatment effects, and marked heterogeneity in gait and postural phenotypes within and across these disorders. Nonetheless, emerging evidence from larger, well-characterized cohorts suggests subtle but measurable gait differences within the LBD spectrum [6, 32]. In a cross-sectional study using wearable sensors, individuals with PDD showed greater motor burden than those with DLB, with higher overall motor severity, lower balance confidence, and increased swing-time and stance-time asymmetry [29]. These parameters differentiated PDD from DLB with moderate accuracy, suggesting that gait variability and balance asymmetry may be sensitive markers of subtype differences. Such distinctions likely reflect differences in onset and progression, with PDD developing after years of motor symptoms and DLB presenting with early cognitive involvement [3]. In contrast, a recent longitudinal study of 157 PDD and 837 DLB patients found no significant difference in the annual rate of motor progression after adjustment for demographic and treatment factors, indicating that once dementia is established, the two follow comparable trajectories of motor decline despite differences in baseline severity and presentation. At dementia diagnosis, however, PDD patients present with more severe motor symptoms and require higher dopaminergic doses than DLB patients, likely reflecting longer preceding motor disease and more pronounced nigrostriatal degeneration [32].

Fall characteristics further distinguish these two subtypes: individuals with DLB experience more frequent, unpredictable, and spontaneous falls than those with PDD, in whom falls are often triggered by turning, gait initiation, or freezing episodes [33]. Recognition of freezing of gait is therefore clinically relevant in patients with LBD, as it contributes substantially to fall risk, may reflect specific fronto-subcortical dysfunction, and is amenable to targeted physiotherapy-based rehabilitation [34]. Moreover, although DLB and PDD exhibit similar rates of motor decline once dementia is established, considerable variability in gait and postural features persists within the LBD spectrum, suggesting that factors beyond clinical subtype contribute to these differences [35]. Recent work has explored the contribution of co-existing AD pathology to this variability. In a biomarker-stratified cohort of LBD patients, higher gait and postural instability scores on the Movement Disorder Society-Unified Parkinson’s Disease Rating Scale Part III were selectively associated with cerebrospinal fluid amyloid-β positivity, whereas tremor, rigidity, and bradykinesia did not differ between biomarker groups. This association persisted after adjusting for total motor severity, suggesting that AD co-pathology may specifically exacerbate gait and postural impairments in LBD [35]. Additional evidence indicates that amyloid-β pathology itself can shape gait phenotypes. In a cohort of non-PD patients presenting with central neurological gait abnormalities, amyloid-positive individuals demonstrated a distinct parkinsonian gait signature, characterized by greater bradykinesia, shorter stride length, and slower gait speed, compared with amyloid-negative patients [36]. These findings suggest that amyloidopathy may contribute to gait impairment and could partly account for the variability observed within the LBD spectrum, particularly in individuals with co-existing AD pathology [37]. The relevance of gait abnormalities may also extend to prodromal LBD, although evidence remains limited. In a pathologically confirmed prodromal DLB cohort, gait disorder and slowness were each reported in approximately one-third of patients and were more frequent than in prodromal AD, while nearly one-third had no parkinsonism at initial presentation [38]. In a community-based study, cognitively unimpaired participants with probable REM sleep behavior disorder, a frequent prodromal feature of Lewy body disorders, had lower gait velocity and cadence and greater double-support, stride-time, and swing-time variability compared with controls [39]. These observations support further longitudinal, biomarker-based studies to determine whether early gait abnormalities have prognostic value in individuals at risk for LBD.

### Cognitive-Motor Interplay within the LBD Spectrum

Few studies have examined dual-task gait testing (walking while performing a simultaneous cognitive task) as a means to assess cognitive-motor interaction in LBD [40]. This paradigm challenges the cognitive component of locomotion and reflects the neural integration of motor and cognitive control [41]. In AD, structural and functional imaging studies show that both rely on shared prefrontal and temporal networks, which may become overloaded when cognitive and motor tasks are performed concurrently, particularly in individuals with reduced cognitive reserve [42, 43]. Comparable behavioral evidence in LBD suggests a similar vulnerability of executive and visuospatial systems during dual-task performance, although direct neuroimaging confirmation remains limited [9]. The degree of gait slowing during dual-task conditions, expressed as the dual-task gait cost, correlates linearly with executive, attentional, and memory deficits [44, 45]. In addition, the study of gait variability, the stride-to-stride fluctuations in walking, offers a complementary insight into cognitive-motor integration. In healthy aging, dual tasking typically reduces gait speed but leaves stride-to-stride variability largely unchanged, a pattern described by Hausdorff [46], who showed that gait rhythmicity remains highly automatic even under substantial cognitive load. In contrast, when automaticity is impaired, as demonstrated in PD individuals, dual tasking increases both gait slowing and temporal variability [47]. Similar dual task-related increases in variability have been observed in behavioural variant of frontotemporal degeneration, where frontal-executive disruption is prominent, highlighting the dependence of gait rhythmicity on intact executive control [48]. This framework is highly relevant to LBD, where early executive dysfunction likely contributes to the marked dual-task interference observed in this population.

While dual-task paradigms effectively capture cognitive-motor interactions, higher dual-task gait costs have been associated with an increased risk of progression from mild cognitive impairment to dementia [44]. In that longitudinal cohort, approximately one-quarter of participants developed dementia over 6 years, predominantly AD, with smaller proportions converting to LBD, frontotemporal, and vascular dementias. These findings underscore the prognostic value of dual-task gait performance as an early marker of neurodegeneration. However, the predominantly amnestic MCI cohort may have masked progression patterns typical of non-Alzheimer dementias such as LBD. In LBD, dual-task paradigms provide additional insight into these distinct motor-cognitive dynamics. LBD participants perform worse under dual-task conditions, with greater difficulty adapting gait as attentional demands increase [40]. This dual-task cost reflects the increased reliance on higher order cognitive processes to maintain gait [49]. From a neurological perspective, walking in adults is gradually consolidated by motor procedural memory, which encodes action rules such that gait becomes more automatic and requires minimal conscious control [50]. This implicit memory system underlies many well-practiced motor routines [51, 52]. In neurodegenerative conditions, particularly AD, procedural memory is relatively preserved compared with declarative memory, enabling patients to perform well-learned motor sequences despite marked episodic memory loss [51, 53]. Beyond procedural memory, other sensorimotor and subcortical systems that support gait tend to remain functional until later disease stages [14, 54]. These include brainstem locomotor circuits, which can sustain basic stepping rhythms even when cortical control is severely impaired [54], habitual motor schemas mediated by the dorsolateral striatum and cerebellum, which enable overlearned walking routines to be triggered by environmental cues with minimal executive input [55], and rhythmic entrainment mechanisms involving cerebellar-premotor networks, which remain responsive to auditory cues and can improve gait timing and stride regularity even in advanced AD [56, 57]. Although described in PD and AD, the role of these mechanisms in LBD remains unexplored; given their relative preservation in other neurodegenerative conditions, similar pathways may help maintain basic gait functions in LBD.

#### Neural Basis of Gait in LBD

LBD affects both cortical and subcortical regions, and its clinical heterogeneity is thought to reflect variation in the regional distribution of alpha-synuclein accumulation and associated co-pathologies [58]. Given that AD co-pathology is present in over 50% of DLB and more than 35% of PDD cases, β-amyloid and related pathology likely contribute to cortical disturbances relevant to gait [59]. Research has largely focused on the neural basis of cognitive dysfunction in LBD, whereas motor disturbances, despite their early onset and role in falls, remain understudied [9, 60].

On the cortical level, gait disturbances in LBD have been linked to structural changes in regions involved in cognition, visuospatial processing, and motor control [38]. A recent voxel-based morphometry study showed that reduced gait speed correlates with gray matter atrophy in motor-related areas such as the primary motor cortex and supplementary motor area, as well as in limbic and associative regions including the anterior and mid-cingulate cortices and the hippocampus [9]. Hippocampal atrophy is typically linked to AD, but postmortem MRI and neuropathologic evidence show it can occur in non-AD dementias, including DLB, indicating that medial temporal atrophy is sensitive but not specific to AD [61]. The hippocampus, traditionally linked to memory, also supports spatial navigation and visuospatial processing, and its atrophy is consistently associated with slower walking in mild cognitive impairment and LBD [30, 62, 63]. Moreover, total gray matter volume correlates with both gait speed and cognitive performance in LBD but not in AD, suggesting disease-specific neuroanatomical substrates of gait impairment [64]. This relationship is particularly evident during dual-task walking, suggesting that limbic and associative cortical regions contribute significantly to cognitively demanding locomotor control [65].

On the subcortical level, structural compromise in regions such as the caudate nucleus has been repeatedly associated with reduced gait speed in LBD [20]. The caudate, part of the striatal system, supports executive motor control and movement initiation through its connections with frontal cortical areas [9, 66]. Its dorsal portion is implicated in spatial working memory and cognitive-motor integration, while the ventral portion is more closely tied to affective and motivational processes [67]. In prodromal to moderate DLB, Botzung et al. [68] showed in a volumetric MRI study that reduced caudate volume, particularly in the left dorsal caudate, correlates with slower processing speed independently of global disease severity, suggesting that basal ganglia pathology contributes to both motor and cognitive gait deficits. In addition, other recent voxel-based morphometry analyses have also identified the cerebellar cortex as a significant correlate of walking speed in DLB [9, 69]. The cerebellum is critical for motor coordination, timing, and predictive control, and for motor-cognitive integration through its reciprocal connections with frontal and parietal association areas [13]. It also supports compensatory mechanisms in PD, where reduced supplementary motor area and basal ganglia activity during locomotion is accompanied by increased cerebellar activation, especially when gait is facilitated by external visual cues. Thus, as neurodegeneration advances, gait reflects a dynamic interplay between cortico-basal ganglia dysfunction and compensatory recruitment of cerebellar and cortical networks. Activity-based interventions, particularly repetitive or dual-task gait training, may enhance these pathways by strengthening cerebellar-cortical coupling, even with dopaminergic loss [70].

Quantitative gait analyses in LBD support the concept that gait disturbances result from disruption of interacting cognitive-motor networks, rather than from isolated motor system degeneration [71]. Mc Ardle et al. [21] showed that in LBD, executive dysfunction and prefrontal cortical involvement accounted for a significant proportion of gait variability, particularly in step time and step length, whereas in AD, gait alterations were primarily related to global cognitive decline. These findings suggest that fronto-striatal disconnection impairs the automatic control of locomotion, leading to increased reliance on higher order executive functions for gait regulation [21, 71]. This shift reflects dysfunction within prefrontal-basal ganglia-cerebellar loops, which normally integrate attentional, planning, and visuospatial processes for efficient motor execution [9, 72]. As these circuits deteriorate, gait becomes less rhythmic and more attentionally demanding, consistent with the observed gait variability, asymmetry, and dual-task cost characteristic of LBD [21, 26]. In this framework, gait disturbances are not merely motor symptoms but manifestations of widespread network-level disruption bridging cognitive and motor systems, aligning with the broader theory of interacting cognitive-motor networks that underpins locomotor control LBD.

#### When Walking Ends: Gait Decline as a Window into End-Of-Life Trajectories in LBD

Individuals with advanced dementia are often treated as a homogeneous group, in disregard to the variability across dementia subtypes [73, 74]. LBD presents unique psychosocial and physical challenges that distinguish it from other dementias, including pronounced fluctuations in attention and alertness, early visual hallucinations, marked sensitivity to antipsychotic medications, and the combination of parkinsonism with autonomic and sleep disturbances [22]. Without disease-modifying therapies, management remains symptomatic and palliative and must adapt to the evolving symptoms, particularly in late-stage and end-of-life phases [75]. Longitudinal studies on autopsy-confirmed LBD cases have shown that individuals with LBD tend to experience a more rapid cognitive and functional decline at the end of life compared to those with AD [76]. However, predicting the transition into the terminal stage of LBD remains difficult, as no widely accepted clinical criteria currently define this phase [77]. Some studies have proposed clinical indicators, suggesting proximity to end of life in LBD, including worsening of cognitive deficits and increased neuropsychiatric symptom severity [76, 78]. Importantly, while cognitive or neuropsychiatric symptoms can be useful indicators of clinical deterioration, they may not sufficiently reflect functional decline or the specific dynamics of the terminal stage [79]. Few studies have examined the trajectories of non-cognitive features, such as physical functioning and dependence, despite their potential prognostic relevance. In a study of elderly hospitalized patients, reduced gait speed, walking distance ≤300 m/day, and impaired standing balance were all significantly associated with increased 100-day mortality [80]. Consistently, in qualitative reports of end-of-life experiences in individuals with DLB, caregivers frequently described a rapid worsening of walking and gait in the weeks preceding death [81]. Late-stage gait decline also reflects non-neurological comorbidities common near the end of life, including systemic illness, frailty, and deconditioning, and thus is not a purely disease-specific trajectory [82].

Gait impairment is a well-recognized hallmark of the aging process [83], and walking speed has been widely used as a proxy for overall health status and as a predictor of mortality risk [83, 84]. However, despite progressive motor decline in LBD, broader locomotor capacity may sometimes remain preserved until relatively late in the disease course [85]. When gait becomes too impaired to serve as a reliable indicator in advanced stages, assessment can shift toward broader measures of mobility that capture residual motor abilities and postural control, such as the capacity to sit, stand, and transition between positions [85]. Although palliative care should ideally be introduced early in dementia trajectories to promote autonomy and goals of care discussions, these patients often do not access palliative care services until the end of life [73]. Within this context, abrupt worsening of walking and gait, often accompanied by increasing instability and falls, may serve as a practical indicator of approaching end of life [86]. Incorporating simple gait assessments into palliative care planning could help clinicians anticipate care needs, reduce fall-related complications, and support timely conversations with families [86].

## Discussion

This review examined distinct gait phenotypes across individuals within the LBD spectrum, as well as in PD and AD. In line with current evidence, gait characteristics differentiate these disorders at comparable mild-to-moderate disease stages [26]. In early AD, gait velocity and stride length are relatively preserved, while increased gait variability and impaired postural control reflect cortical cognitive dysfunction, particularly executive and attentional control [31]. This interpretation is supported by work showing that executive dysfunction contributes to gait instability and fall risk in AD, whereas stride-time variability is more strongly associated with frontal dysfunction in frontotemporal dementia [48, 87].

Individuals with AD generally show relatively preserved gait velocity and stride length and preserved balance, consistent with cortical rather than extrapyramidal involvement. PD presents with a hypokinetic, narrow-based gait marked by reduced stride length, prolonged stance, and diminished swing phase [14]. Gait deficits are ultimately most pronounced in LBD compared to PD, where slower speed, shorter stride, and greater stride-to-stride variability were accompanied by poorer balance and dual-task performance [21, 26]. Within the LBD spectrum, motor performance in PDD and DLB becomes broadly comparable once cognitive decline reaches similar levels, although differences in postural balance remain inconsistently reported, largely due to small sample sizes [88]. Across LBD and PD, gait alterations correlate with global cognition and executive function, consistent with observations in other dementia subtypes such as AD and vascular dementia [89, 90]. These associations support gait variability and dual-task costs as potential markers of cognitive-motor coupling and overall disease progression [88]. Recent volumetric neuroimaging studies indicate that gait-cognition interactions rely on overlapping fronto-subcortical and cortical networks across dementia types [91]. At the same time, comparative gait studies have identified distinct gait signatures, reflecting disease-specific contributions to motor control [48]. Together, these findings suggest that shared neural substrates underlie cognitive-motor coupling, while disorder-specific alterations modulate gait by subtype. In DLB, this is reflected by a strong association between gait and global cognition, where slower velocity and shorter stride parallel cognitive decline [9]. Neuroimaging evidence further indicates that gait slowing corresponds to gray matter atrophy within the parietal, temporal, and insular cortices - regions involved in visuospatial and attentional control [9]. This relationship may be particularly pronounced in DLB due to its common dual pathological burden of both α-synuclein and β-amyloid [7]. Abnormal α-synuclein aggregation disrupts motor control through basal ganglia circuits and extends along limbic pathways to the prefrontal cortex, impairing executive function. Coexistence of these pathologies likely amplifies the gait-cognition coupling observed in DLB compared with pure AD or PD [88].

Cognitive measures have traditionally served as anchors for clinical staging and prognostication in dementia, yet they may be less reliable in more advanced stages, when non-cognitive symptoms, such as attentional fluctuations, visual hallucinations, parkinsonism-related slowing, or daytime somnolence, interfere with standardized testing [22]. In DLB, cognitive decline progresses more rapidly than in AD, and neuropsychiatric manifestations tend to emerge earlier in the disease course [92, 93]. Furthermore, patients with DLB have shorter survival after diagnosis compared with those with AD or other dementia types, with mean survival ranging from 3.2 to 7.2 years [93]. Although this accelerated course is defined mainly by cognitive decline, cognitive and motor deterioration may not progress in parallel. In a large community-based cohort without dementia at baseline, Oveisgharan et al. [1] showed that gait and cognitive decline can follow distinct trajectories, with cognition often declining earlier and gait deteriorating later or independently. In this context, gait performance offers an additional dimension for staging disease severity in LBD, integrating motor and cognitive decline. Another longitudinal study found mobility decline steeper in non-Alzheimer dementias than in AD despite comparable cognitive severity, suggesting that gait may better capture disease burden and predict loss of independence [94]. LBD provides a distinctive framework for exploring the prognostic value of gait, as motor and postural disturbances emerge early and remain central throughout the disease course. Gait impairments are nearly universal in LBD and are strongly associated with falls, loss of independence, and increased mortality [83]. These features make gait a compelling candidate marker for advanced disease stages and a more stable anchor for clinical staging in late phases and promoting concomitant palliative care expertise [95]. The integration of digital technologies into clinical research is further redefining how motor function is quantified in neurodegenerative disorders [96]. Recent studies in early PD have shown that digital biomarkers derived from wearable and smartphone technologies can capture longitudinal changes in gait and tremor, distinguishing patients from controls and detecting subtle progression beyond conventional clinical scales [97]. However, in LBD, assessing motor and other non-cognitive symptoms remains challenging, as cognitive fluctuations and attentional deficits may compromise reliable self-reporting, and substantial interobserver variability persists with conventional motor rating instruments [98]. Taken together, this review underscores the need to view gait not merely as a functional parameter but as a window into neurological function that may follow its own trajectory, even as cognition declines.

## Conclusion

Refining disease progression measures in LBD remains a critical need. Gait impairment in LBD reflects the complex interplay of cognitive-motor networks that underpin locomotor control, representing an additional and clinically relevant dimension of disease severity. Because gait and cognition do not decline in parallel, gait may serve as a complementary and more stable indicator of progression that integrates both motor and cognitive components of decline, including in advanced stages where cognitive measures lose discriminative value. In this context, monitoring gait can also inform supportive care planning by anticipating mobility decline and fall risk. Ultimately, acknowledging gait as a key marker of neurological decline offers a more comprehensive understanding of functional deterioration and end-of-life trajectories in LBD, where walking may, at times, outlast cognition.

## Conflict of Interest Statement

Gilles Allali was a member of the journal’s Editorial Board at the time of submission. Patrick Stancu, Frédéric Assal, Anna E. Goodheart, Lisa Hentsch, and Caroline Hertler report no disclosure.

## Funding Sources

This study was not supported by any sponsor or funder.

## Author Contributions

P.S. drafted and edited the manuscript and created the figures. L.H. and C.H. made a substantial contribution to the concept of the article. A.E.G., L.H. edited the manuscript. A.E.G., G.A., and F.A. performed critical revision of the manuscript.
